# Worse treatment response to neoadjuvant chemoradiotherapy in young patients with locally advanced rectal cancer

**DOI:** 10.1186/s12885-020-07359-2

**Published:** 2020-09-05

**Authors:** Yiyi Zhang, Liangliang Yan, Yong Wu, Meifang Xu, Xing Liu, Guoxian Guan

**Affiliations:** 1 Department of Colorectal Surgery, The First Affiliated Hospital of Fujian Medical University, Fuzhou, China; 2 Department of Cardiac Surgery, Fujian Medical University Union Hospital, Fuzhou, China; 3 Department of Pathology, Fujian Medical University Union Hospital, Fuzhou, China

**Keywords:** Age, pCR, Prognosis, LARC, CD133

## Abstract

**Background:**

To evaluate the impact of age on the efficacy of neoadjuvant chemoradiotherapy (NCRT) in patients with locally advanced rectal cancer (LARC).

**Method:**

LARC patients undergoing NCRT and radical surgery from 2011 to 2018 were divided into young (< 40 years) and old (≥40 years) groups. Multivariate analyses were performed to identify predictive factors for pathological complete response (pCR). Predictive nomograms and decision curve analysis were used to compare the models including/excluding age groups. Immunohistochemical analysis was performed to detect CD133 expression in LARC patients.

**Result:**

A total of 901 LARC patients were analyzed. The young group was associated with poorly differentiated tumors, more metastatic lymph nodes, higher perineural invasion, and a lower tumor regression grade (*P* = 0.008; *P* < 0.001; *P* < 0.001; *P* = 0.003). Logistic regression analysis demonstrated that age < 40 years (HR = 2.190, *P* = 0.044), tumor size (HR = 0.538, *P* < 0.001), pre-NCRT cN stage (HR = 0.570, *P* = 0.036), and post-NCRT CEA level (HR = 0.877, *P* = 0.001) were significantly associated with pCR. Predictive nomograms and decision curve analysis demonstrated that the predictive ability of models including the age group was superior to that of models excluding the age group. Higher CD133 expression was more common in young LARC patients.

**Conclusion:**

Young patients with LARC were associated with lower pCR rates following NCRT. The ability of the predictive model was greater when based on the age group. Young LARC patients were associated with a higher CD133+ tumor stem cell burden, which contributed to the lower pCR rates.

## Background

Colorectal cancer (CRC) is the third most common cancer and the second leading cause of cancer-related mortality in the USA [1]. CRC is generally thought to be a malignancy affecting the elderly  patients. Over the last two decades, the incidence of CRC has increased in young individuals, especially those aged under 40. However, most studies focus on older CRC patients, especially the elderly (> 70 years) [2, 3]. Few studies have focused on the impact of young age (< 40 years) in CRC patients. In contrast to CRC in the elderly, young patients present at a more advanced tumor stage, with a more aggressive pathological subtype, and poor prognosis [4–6]. The increasing prevalence in CRC patients aged < 40 years highlights a genuine need to better understand this disease in such patients.

Neoadjuvant chemoradiotherapy (NCRT) and radical resection have become the standard treatment for patients with locally advanced rectal cancer (LARC). The well-documented benefits of this multimodality include a higher probability of tumor downsizing and downstaging, improved tumor resectability and sphincter preservation, and better local tumor control [7–9]. Approximately 10–30% of patients will achieve a pathological complete response (pCR), heralding an excellent prognosis due to low rates of local and distant recurrence [7, 10, 11]. Major efforts have been devoted to evaluating the influence of age on the efficacy and compliance of NCRT. While most studies were focused on old patients, few studies have focused on young LARC patients. Considering the aggressive tumor biology in young patients, we hypothesize that the efficacy of NCRT is reduced in young patients. Several recent studies have evaluated the impact of age on the efficacy of adjuvant chemotherapy in patients with rectal cancer [2, 12, 13]. Cancer stem cells (CSCs) have the capacity for multipotency and self-renewal and may be responsible for neoplasm formation, metastasis, recurrence, and therapeutic resistance [14–16]. Thus, we hypothesize that the early onset is due to higher malignancy cells, such as CSCs in young patients. Moreover, CSCs also indicate a poorer response to NCRT in LARC patients. However, data regarding the association of young age with treatment response to NCRT are scarce, and the influence of young age on the efficacy of NCRT in LARC patients remains unclear.

In this context, the present study was conducted to compare the efficacy of NCRT in young (< 40 years) and old (≥40 years) patients with LARC in terms of tumor response. Additionally, we further investigated the relationship between young age and CSC (CD133 expression) frequency in LARC patients following NCRT.

## Patients and method

### Patient eligibility

**Fig. 1:**
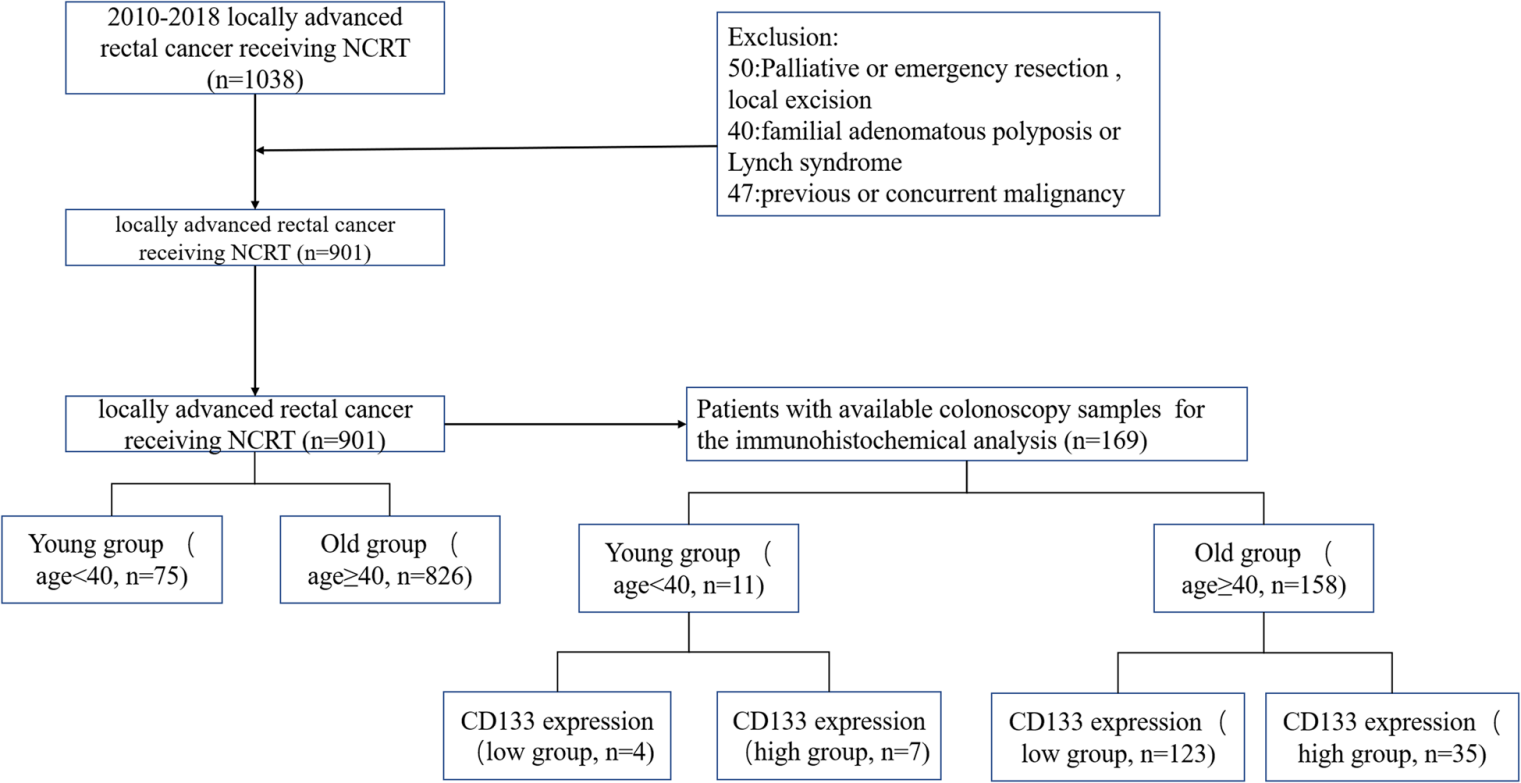
Patient flow diagram

A retrospective study based on our prospectively maintained database was performed. LARC patients who underwent NCRT and radical resection between 2011 and 2018 were identified. The inclusion criteria were as follows: 1) clinical stage II or III (cT3/4 or cN1/2) disease; 2) pathologically proven rectal adenocarcinomas; and 3) tumors located within 12 cm from the anal verge. Exclusion criteria included: 1) previous of malignancies or concurrent with malignancies; 2) patients who underwent emergent surgery, palliative resection, or local excision. Finally, a total of 901 LARC patients were included. Since the majority of patients were pathologically confirmed by endoscopic biopsy when admitted to our hospital (a tertiary referral hospital), colonoscopy samples from only 169 patients were available for the immunohistochemical analysis. This study was approved by the Institutional Review Board (IRB) of Fujian Medical University Union Hospital. A patient flow diagram was shown in Fig 1.

### Treatment protocol

Comprehensive assessments for tumor staging of patients were performed by a digital rectal examination, colonoscopy, chest radiography, abdominopelvic magnetic resonance imaging (MRI), and/or transrectal ultrasound (ERUS). Preoperative long-course radiotherapy consisted of a total dose of 45 Gy to the pelvis, delivered in 25 fractions for 5 consecutive weeks (180 cGy per fraction, 5 days a week), followed by a boost of 5.4 Gy to the primary tumor. Preoperative concurrent chemotherapy was initiated on the first day of radiotherapy by using 5-FU plus oxaliplatin (FOLFOX) or capecitabine plus oxaliplatin (CapeOX).

Surgical operation was performed at an interval of 6–8 weeks after the completion of radiation. Surgical resection for rectal cancer was performed according to the principle of total mesorectal excision (TME) and high ligation of the inferior mesenteric artery. The surgical procedure consisted of low anterior resection (LAR), abdominoperineal resection (APR), or Hartmann’s procedure. About 3–4 weeks after surgery, postoperative adjuvant chemotherapy was administered to patients (using FOLFOX or CapeOX) for 6 months.

### Definitions

Tumor response to NCRT was graded according to the tumor regression grade (TRG) system [17]. pCR was defined as the absence of viable tumor cells in either the primary tumor site or the resected lymph nodes. Postoperative morbidity was classified according to the Clavien-Dindo classification, grades I-II was considered as minor complications, and grades III-V as major complications. Tumor size was divided according to the quartile intervals to make it more applicable in clinical practice (≤1.8 cm, 1.9–3.1 cm, ≥3.2 cm). Perioperative mortality was defined as any death within 30 days of surgery or occurring in the hospital.

### Immunohistochemical analysis

CD133 (Affinity Biosciences, AF5120, Polyclonal, 1:100) protein expression in specimens obtained before NCRT in 169 LARC patients was measured using the immunohistochemical streptavidin-biotin complex method (Fig. 3e, f, g, h) [18]. Phosphate-buffered saline (PBS) was used as the negative control and the image of the positive control from GE Healthcare Life Sciences. Immunoreactivity was scored by semi-quantitative analysis, and the fields were randomly selected in five directions (up, center, down, left, and right) under high magnification (× 400). The color was determined based on the intensity score as follows: 0 (no staining), 1 (light yellow), 2 (brown), and 3 (deep brown). The percentage of positive cells was scored as 0 (< 5%), 1 (5–25%), 2 (25–50%), 3 (50–75%), and 4 (> 75%). The mean value was calculated for each case with the aforementioned scoring methods and the final score was obtained by multiplying these two scores. All analyses were performed in a double-blinded manner.

### Statistical analysis

Statistical analysis was performed using SPSS version 23.0 (SPSS INC., Chicago, USA) and R software packages, version 3.5.1 (The R Foundation for Statistical Computing, http://www.r-project.org/). The categorical variables were presented as frequencies and percentages and assessed using the Chi-squared (*χ*^*2*^) test or Fisher’s exact test. The continuous variables were described as means ± standard deviation and assessed using Student’s *t*-test. All significant variables in the univariate analysis were entered into a Logistic regression model to identify predictors of pCR. Based on the multivariable analysis, a predictive nomogram was developed using the R project. The performance of the nomogram was evaluated by calculating the Harrell’s concordance index (c-index). Decision curve analysis (DCA) was performed to evaluate the clinical utility of the model for pCR. DCA is a method for evaluation and comparison of the predictive value between different prediction models [19, 20]; therefore, this method was used to evaluate the clinical utility of the model for pCR. The x-axis of the DCA represents the percentage of threshold probability, and the y-axis represents the net benefit of the predictive model. The net benefit was calculated according to the following formula: Net benefit = (true positives/n) − (false positives/n) * (pt/(1 − pt). “Number high risk” indicated the number of patients classified as positive (high risk) by a model including age group according to various threshold probabilities. “Number high risk with the event” was the true positive patient number according to various threshold probabilities. The optimal cut-off values for CD133 expression were calculated and determined by using the X-tile program (http://www.tissuearray.org/rimmlab/), a new bio-informatics tool for biomarker assessment and outcome-based cut-point optimization, which identified the cut-off with the minimum *p* values from log-rank χ2 statistics in terms of OS [21]. The optimal cut-off score was identified as 11 for CD133. Thus, we defined CD133 expression score ≤ 11 as low expression, and > 11 as high expression. Survival outcomes were assessed using the Kaplan–Meier method and log-rank test. *P* < 0.05 was considered to indicate statistical significance.

## Results

### Patient characteristics

A total of 901 LARC patients were eligible for our analysis. Among them, 75 (8.3%) were assigned to the young group and 826 (91.7%) patients were assigned to the old groups. The median ages in the two groups were 34.3 and 58.1 years, respectively. Additionally, the old group was associated with a higher American Society of Anaesthesiology (ASA) grade (*P* < 0.05). As shown in Table 1, there were no significant differences between the two groups in terms of sex, the interval between NCRT and surgery, distance from the anal verge, clinical T stage, clinical N stage, pre-NCRT CA19–9 level, pre-NCRT CEA level, post-NCRT CA19–9 level, and post-NCRT CEA level (all *P* > 0.05),

**Table 1:** Patient characteristics in patients with LARC after NCRT

Characteristics	Young group (*n* = 75)	Old group (*n* = 826)	*p* value
Sex (%)			0.527
Male	52 (69.3)	537 (65.0)	
Female	23 (30.7)	289 (35.0)	
Age (years)	34.3 ± 4.1	58.1 ± 9.4	**< 0.001**
ASA score (%)			**< 0.001**
1	75 (100)	603 (73.0)	
2	0	209 (25.3)	
3	0	14 (1.6)	
Distance from the anal verge (cm)	6.4 ± 2.5	6.3 ± 2.3	0.684
Time interval between CRT and surgery (weeks)	9.1 ± 1.4	9.4 ± 2.9	0.468
Clinical T stage (%)			0.173
T1	1 (1.3)	2 (0.2)	
T2	0	18 (2.2)	
T3	25 (33.3)	317 (38.4)	
T4	49 (65.3)	489 (59.7)	
Clinical N stage (%)			1.000
N0	7 (9.3)	79 (9.5)	
N+	68 (90.7)	747 (90.5)	
Pre-NCRT CEA level (%)			0.562
< 5.0 ng/ml	25(33.3)	283 (34.3)	
≥ 5.0 ng/ml	24(32.0)	219 (26.5)	
Unknown	26(34.7)	324 (39.2)	
Pre-NCRT CA19–9 level (%)			0.641
< 39.0 U/ml	40 (53.3)	430 (52.1)	
≥ 39.0 U/ml	9 (12.0)	76 (9.2)	
Unknown	26(34.7)	320 (38.7)	
Post-NCRT CEA level (%)			0.115
< 5.0 ng/ml	67 (89.3)	672 (81.4)	
≥ 5.0 ng/ml	8 (10.7)	154 (18.6)	
Post-NCRT CA19–9 level (%)			0.320
< 39.0 U/ml	68 (90.7)	775 (93.8)	
≥ 39.0 U/ml	7 (9.3)	51 (6.2)	

*LARC* Locally advanced rectal cancer; *NCRT* Neoadjuvant chemoradiotherapy; *ASA* American Society of Anesthesiologists; *CRT* Chemoradiotherapy; *CEA* Carcinoembryonic Antigen; *CA19–9* Carbohydrate Antigen 19–9

### Perioperative, pathological and survival outcomes

No significant differences were observed between the two groups in terms of estimated blood loss, operation time, surgical approach, peri-NCRT complication rates, and sphincter-saving procedure (all *P* > 0.05, Table 2). There were no significant differences between the two groups in terms of postoperative hospital stay and postoperative complications (*P* = 0.124, *P* = 0.736, respectively). Similarly, the chemotherapy regimen did not differ between the two groups (*P* = 0.461). Compared to the young group, mucinous or signet ring cell carcinoma, (17.3% vs. 7.6%, *P* = 0.008) or poorly differentiated tumors (24.3% vs. 9.0%, *P* < 0.001) were more common in the old group. Moreover, the young group was associated with a higher TRG (*P* = 0.003), as well as a higher rate of perineural invasion (17.3% vs. 6.5%, *P* = 0.002). Pathological TNM stage and pathological type were similar in both groups (*P* = 0.957, *P* = 0.936). Similarly, there were no differences between the two groups in terms of vascular invasion and tumor size did (*P* = 0.346, *P* = 0.069). A positive circumferential resection margin (CRM) was observed in one patient (1.3%) in the young group, 10 patients (1.2%) in the old group, with no significant difference between the two groups (*P* = 1.000). Moreover, Kaplan-Meier curve analysis demonstrated that young patients were associated with poorer prognosis in LARC patients following NCRT. The 3-year OS rate in the old group (≥40 years) was significantly higher than that in the young group (< 40 years) (88.3% vs. 71.6%; *P* = 0.01, Fig. 3m). Moreover, the 3-year DFS rate for the old group (≥40 years) was higher than that in the young group (< 40 years) (83.8% vs. 68.6%; *P* = 0.204, Fig. 3l).

**Table 2:** Operative and postoperative outcomes in patients with LARC after NCRT

Characteristics	Young group (*n* = 75)	Old group (*n* = 826)	*p* value
Operative time (min)	232.5 ± 81.2	225.8 ± 64.0	0.394
Estimated blood loss (ml)	77.7 ± 74.2	88.9 ± 105.5	0.372
Surgery approach (%)			0.882
Laparoscopic	49 (65.3)	549 (66.5)	
Open	18 (24.0)	198 (23.9)	
Robotic	8 (10.7)	79 (9.6)	
Postoperative hospital stay (days)	7.8 ± 3.4	8.8 ± 5.5	0.124
Postoperative complications (%)	12 (16.0)	122 (14.9)	0.736
30 days readmission (%)	1 (1.3)	5 (0.6)	0.407
Peri-CRT complications^a^	25 (33.3)	259 (31.4)	0.699
Major	1 (1.3)	23 (2.8)	0.741
Sphincter-saving procedure (%)	66 (88.0)	721 (87.4)	1.000
Pathological type (%)			0.936
Ulcering	72 (96.0)	796 (96.7)	
Expanding	1 (1.3)	11 (1.2)	
Infiltrating	2 (2.7)	19 (2.1)	
Histopathology (%)			**0.008**
Adenocarcinoma	62 (82.7)	763 (92.4)	
Mucinous or signet ring cell carcinoma	13 (17.3)	63 (7.6)	
Tumor differentiation (%)			**< 0.001**
Well to moderately differentiated	57 (75.7)	752 (91.0)	
Poorly differentiated and others	18 (24.3)	74 (9.0)	
Chemotherapy regimen (%)			0.461
FOLFOX/CapeOX	33 (44.0)	326 (39.5)	
Capecitabine	42 (56.0)	500 (60.5)	
Lymph nodes retrieved	19.3 ± 13.1	12.0 ± 6.2	**< 0.001**
Metastatic lymph nodes	2.2 ± 6.1	0.7 ± 2.0	**< 0.001**
CRM involvement (%)	1 (1.3)	10 (1.2)	1.000
Tumor size (cm)			0.069
≤ 1.8 cm	17(22.7)	212 (25.7)	
1.9–3.1 cm	31 (41.3)	416 (50.4)	
≥ 3.2 cm	27 (36.0)	198 (24.0)	
Pathological TNM stage (%)			0.957
0	9 (12.0)	183 (22.2)	
I	14 (18.7)	205 (24.8)	
II	16 (21.3)	217 (26.3)	
III	31 (41.3)	179 (21.7)	
IV	5 (6.7)	42 (5.2)	
TRG grade (%)			**0.003**
0	9 (12.0)	183 (22.2)	
1	20 (26.7)	272 (32.9)	
2	33 (44.0)	313 (37.9)	
3	13 (17.3)	58 (7.0)	
Perineural invasion (%)	13 (17.3)	54 (6.5)	**0.002**
Vascular invasion (%)	4 (5.3)	29 (3.5)	0.346
Adjuvant chemotherapy (%)			0.484
Complete	44 (58.7)	427 (51.7)	
Decreased complete	9 (12.0)	87 (10.5)	
Refuse	2 (2.7)	44 (5.3)	
Unknown	20 (26.7)	268 (32.4)	

^a^ Some patients experienced more than one complication, and categorized as

*NCRT* Neoadjuvant chemoradiotherapy; *CRM* Circumferential resection margin; *TRG* Tumor regression grade

### Association between young age and pCR

To explore the association between young age and treatment response to NCRT, we identified predictive factors for pCR by Logistic regression analysis. In the univariate analysis, tumor size (≤1.8 cm vs. 1.9–3.1 cm OR = 6.764, *P* < 0.001; vs. ≥3.2 cm OR = 2.022, *P* = 0.007), age (≥40 years vs. < 40 years, OR = 2.087, *P* = 0.044), pre-NCRT clinical T stage (OR = 0.731, *P* = 0.031), pre-NCRT clinical N stage (OR = 0.550, *P* = 0.016), distance from the anal verge (OR = 0.919, *P* = 0.018), and post-NCRT CEA (OR = 0.381, *P* < 0.001) were significantly associated with pCR in LARC patients. In the multivariate analysis, tumor size (≤1.8 cm vs. 1.9–3.1 cm OR = 6.764, *P* < 0.001; vs. ≥3.2 cm OR = 2.022, *P* = 0.007), age (≥40 years vs. < 40 years, OR = 2.382, *P* = 0.027), pre-NCRT clinical N stage (OR = 0.560, *P* = 0.029), and post-NCRT CEA (OR = 0.873, *P* = 0.001) were independent predictive factors for pCR in LARC patients (Table 3).

**Table 3:** Univariate and multivariate analysis of predictive factors for pCR in LARC patients (*n* = 901)

Variables	Univariate analysis			Multivariate analysis		
	HR	95% CI	*p* value	HR	95% CI	*p* value
Sex (male vs. female)	1.351	0.973–1.875	0.073			
Age (≥40 years vs. < 40 years)	2.087	1.020–4.269	**0.044**	2.382	1.105–5.137	**0.027**
ASA	0.964	0.686–1.353	0.831			
Distance from the anal verge	0.919	0.856–0.986	**0.018**	0.949	0.878–1.025	0.181
Tumor size						
≤ 1.8 cm	Reference	Reference	**< 0.001**	Reference	Reference	**< 0.001**
1.9–3.1 cm	6.764	4.019–11.385	**< 0.001**	5.806	3.412–9.878	**< 0.001**
≥ 3.2 cm	2.022	1.212–3.373	**0.007**	1.853	1.101–3.119	**0.020**
Time interval between NCRT and surgery	1.041	0.988–1.097	0.131			
Pre-NCRT cT stage	0.731	0.553–0.967	**0.031**	0.816	0.607–1.097	0.177
Pre-NCRT cN stage	0.550	0.338–0.895	**0.016**	0.560	0.332–0.943	**0.029**
Post-NCRT CEA level	0.381	0.224–0.647	**< 0.001**	0.873	0.805–0.946	**0.001**
Post-NCRT CA19–9 level	0.492	0.219–1.101	0.084			
Chemotherapy regimen	1.083	0.783–1.499	0.630			
Radication dose reduction	0.978	0.360–2.653	0.965			
NCRT complications	0.900	0.641–1.264	0.542			

*pCR* Pathological complete response; *NCRT* Neoadjuvant chemoradiotherapy; *HR* Hazard ratio; *CI* Confidential interval; *ASA* American Society of Anesthesiologists; *CEA* Carcinoembryonic Antigen; *CA19–9* Carbohydrate Antigen 19–9

### Predictive nomogram for pCR and decision curve analysis

**Fig. 2:**
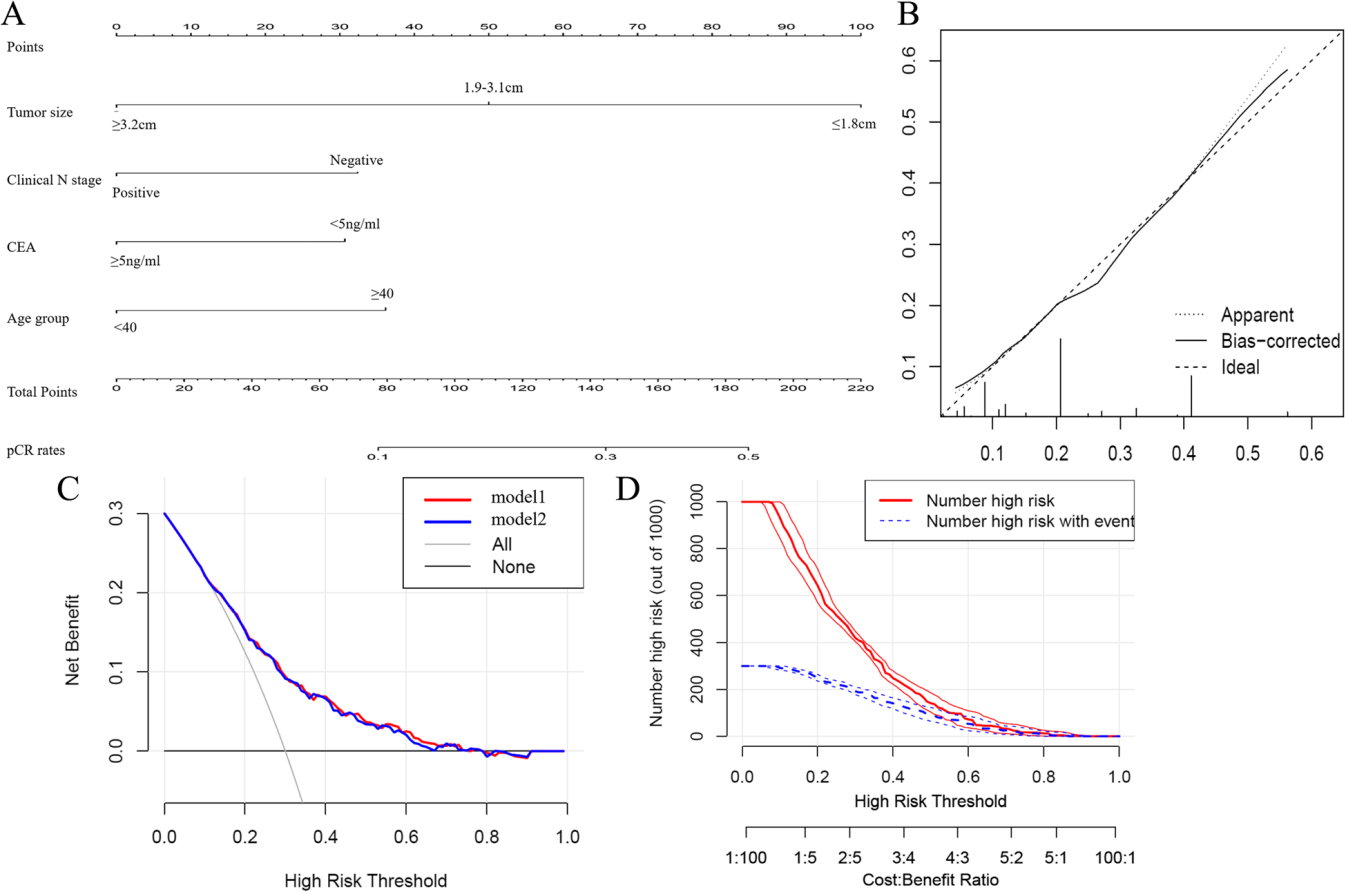
Construction of the models for prediction of pCR rates. (**a**) Nomogram developed for prediction of pCR. Calibration plots in the internal (**b**) validation cohort for pCR. (**c**) Decision curve analysis for pCR. (**d**) Clinical impact curve for the risk model. Of 1000 patients, the red solid line shows the total number of patients deemed to be at high risk for each risk threshold. The blue dashed line shows how many of those would be true positives

By incorporating the significant determinants in the Logistic regression analysis, we developed a predictive nomogram for pCR in LARC patients after NCRT as shown in Fig. 2a. The c-index of the nomogram was 0.70 (95% CI 0.70–0.71). The calibration curve (Fig. 2b) presented good statistical performance upon internal validation between predicted and actual pCR rates. DCA was used to evaluate the performance of the nomogram. As shown in Fig. 2c, the model including age group provided more predictive power than either the pCR scheme or the non-pCR scheme. The clinical impact curve (Fig. 2d) shows the prediction of risk stratification of 1000 patients using a resampling bootstrap method.

### The association of CD133 expression with survival, pCR rate, and young age

**Fig. 3:**
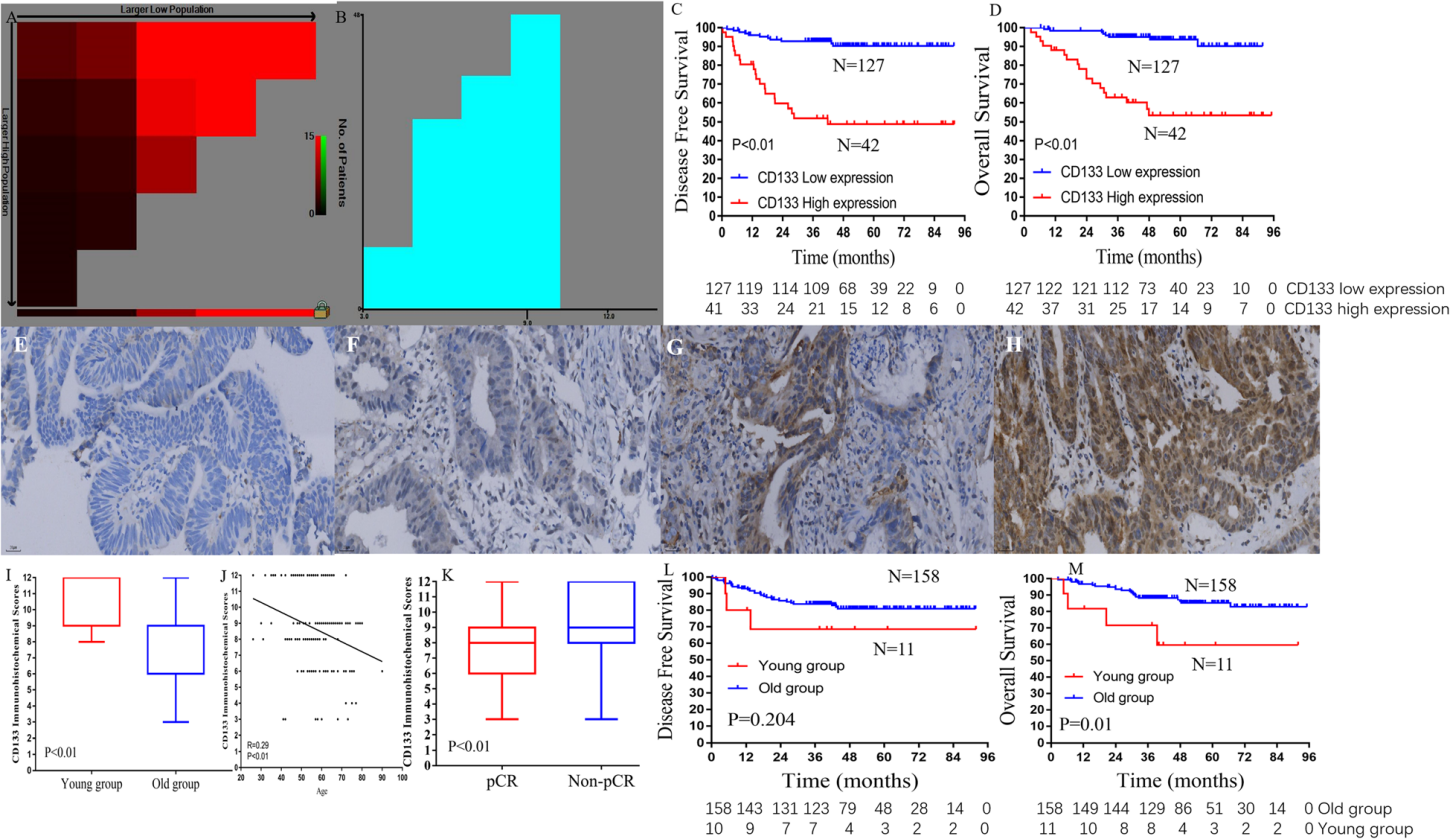
CD133 expression was associated with age and prognosis of LARC patients. (**a**) and (**b**) Cut-off points for CD133 expression determined by the X-tile program. X-tile analysis divided the entire cohort into the training (shown in the upper-left quartile of A) and matched validation sets (shown on the bottom X-axis of A) based on patient survival data. The black dot in the validation set represents the exact cut-off value for CD133 expression. The entire cohort was divided into low (blue) and high (gray) NLR count groups based on the optimal cut-point, as is shown on a histogram of the entire cohort (**b**). (**c**) Kaplan–Meier curve of disease-free survival and (**d**) overall survival for the optimal cut-point of the CD133 expression. Representative figures of expression of CD133 in rectal cancer tissues (10 × 40), (**e**) Negative (−), (**f**) Weakly positive (+), (**g**) Positive (++), (**h**) Strongly positive (+++). (**i**) The CD133 expression scores in the young and old groups. (**j**) The correlation analysis of CD133 expression score and patient age. (**k**) The CD133 expression scores in the pCR and non-pCR groups. (**l**) Kaplan–Meier curve of disease-free survival and (**m**) overall survival for the young and old group

We further investigated the reason for the lower pCR rates in young LARC patients by examining CSC frequency (based on CD133 expression, Fig. 3e, f, g and h). A total of 169 LARC patients were eligible for immunohistochemical analysis. Since the CD133 expression scores were continuous variables, the X-tile program was utilized to identify the optimal cut-off points for determining the greatest actuarial survival difference. Using this method 11 was identified as the cut-off value for CD133 expression (Fig. 3a and b). Based on these cut-off points for CD133 expression, we divided the cohort into low (*n* = 127) and high (*n* = 42) subgroups in terms of OS and DFS.

Higher CD133 scores were associated with poorer prognosis in LARC patients following NCRT. The 3-year OS rate for the low CD133 group was significantly higher than that in the high CD133 group (95.1% vs. 62.9%; *P* < 0.01, Fig. 3d). Moreover, lower CD133 scores were correlated with improved DFS (Fig. 3c). The 3-year DFS rate for the low CD133 group was significantly higher than that in the high CD133 group (92.8% vs. 50.7%; *P* < 0.01). As shown in Fig. 3i, young patients had a higher CD133 expression score compared to that of old patients (*P* < 0.01). Moreover, correlation analysis demonstrated that CD133 expression was associated with patient age (*P* < 0.01, Fig. 3j). Moreover, we examined the CD133 expression in the pCR and non-pCR groups, the results demonstrated that the CD133 expression value in the pCR group was significantly lower than in the non-pCR group (*P* < 0.01, Fig. 3k). Taken together, these findings indicated that young LARC patients were associated with a higher CD133+ CSC burden, which might contribute to the lower pCR rates.

## Discussion

Age is an important factor affecting the efficacy of NCRT in rectal cancer patients. Few studies have focused on young LARC patients following NCRT. In the present study, we explored the efficacy of NCRT in young (< 40 years) and old (≥40 years) LARC patients. The results demonstrated a lower pCR rate in young LARC patients compared with that in old patients, without affecting postoperative complications. For the first time, our study demonstrated that young patients have a higher proportion of CSCs (CD133+), which might contribute to the lower pCR rates.

Young LARC patients often present with aggressive pathological features and advanced stage compared with older patients [4–6]. Additionally, aggressive pathological features could result in a poorer response to NCRT [22–28]. Our results are consistent with those reported by Li et.al [29], in which analysis of the Surveillance, Epidemiology, and End Results (SEER) population-based database revealed that young patients had a better prognosis than old patients. However, the prognosis was inconsistent with the pathological results in their study, because young patients had more aggressive pathological features compared with old patients. This discrepancy may be explained that by the lack of cancer therapy information, including neoadjuvant and adjuvant treatment, and quality of surgery, in the SEER database, all of which are factors that play an important role in patient survival outcome. When treating young LARC patients with rectal cancer, it is important to determine how aggressive the tumor is, so that patients can be informed about the advantages and disadvantages of the treatment. In our study, we demonstrated that young LARC patients displayed poorer pathological features, such as a higher probability of mucinous or signet ring cell and poorly differentiated tumors, which is in accordance with the findings of previous studies [23, 24, 26]. These results indicate that young LARC patients have more severe pathological features than those of older patients.

Age is an important factor for survival benefits and the risk of complications in cancer patients following CRT. To clarify the influence of age on tumor response to NCRT in LARC patients, we performed a logistic regression analysis. pCR has been proposed as a surrogate endpoint for the efficacy of NCRT and oncological outcome in LARC patients. Our results demonstrated that young age is an independent predictive factor for pCR in LARC patients, as well as tumor size, pre-NCRT clinical N stage, and post-NCRT CEA level. Our results also indicated that young LARC patients have poor responses to NCRT, in terms of lower pCR rates. Therefore, more chemotherapy treatment options could be considered in the NCRT regimens in young LARC patients to increase the pCR rate. We further developed a nomogram for predicting pCR to facilitate the decision-making regarding organ-preserving strategies. Our results showed that decisions based on the pCR predictive nomogram yielded more favorable clinical consequences than the treat-all patient and treat-none schemes, even given an extremely small probability threshold. Additionally, the nomogram including age group had superior predictive capability than the nomogram excluding age group.

CSCs are the tumor-initiating cells that are responsible for tumorigenesis [30, 31]. Accumulating evidence has demonstrated that CSCs contribute to resistance to either chemotherapy or radiotherapy in various cancers, including rectal cancer [32–34]. It has been reported that cells expressing CD133, which is a putative marker of CSCs, are more resistant to radiochemotherapy than CD133- tumor cells in rectal cancer [32, 33]. Thus, CD133 expression in the tumor cells was detected as a CSC biomarker in LARC patients. However, the association between CSCs and treatment response in young LARC patients remains unclear. Herein, we hypothesized that young LARC patients have more CSCs, resulting in the resistance to NCRT. In this study, we demonstrated higher CD133 expression in cancer tissue of young LARC patients before NCRT compared with that in the old group. Additionally, higher CD133 expression was correlated with a worse prognosis. Together, these findings suggest that young LARC patients have a higher CD133+ CSC burden, contributing to the lower pCR rates. Nevertheless, prospective studies with large sample sizes are required to confirm the role of CD133 in resistance to NCRT.

Several limitations of our study should be noted. First, our retrospective study was subject to potential selection bias. Second, age-related comorbidities, such as the Charlson comorbidity index, were not evaluated due to the lack of adequate data. Third, the impact of gene profiling on response to NCRT was not assessed owing to the lack of complete medical records in some cases. Fourth, CD133 expression was assessed only in some patients owing to the lack of adequate pretreatment specimens. Nevertheless, our study adds to our understanding of the impact of young age on the efficacy of NCRT in patients with LARC.

## Conclusions

In this cohort study of 901 LARC patients treated at a single high-volume cancer center, young age (< 40 years) was identified as a significant determinant for predicting pCR in LARC patients following NCRT. Moreover, for the first time, we have demonstrated that young patients have a higher proportion of CSCs (CD133+) than older patients. Larger-scale prospective studies are warranted to confirm our findings.

## Data Availability

The data generated or analysed during this study are available from the corresponding author upon reasonable request.

## Abbreviations

NCRT: Neoadjuvant chemoradiotherapy
LARC: Locally advanced rectal cancer
CRC: Colorectal cancer
TME: Total mesorectal excision
CEA: Carcinoembryonic antigen
MRI: Magnetic resonance imaging
pCR: Pathological complete response
ASA: American society of anesthesiology
CRM: Circumferential resection margin
HR: Hazard ratio
DFS: Disease free survival
